# Preload dependence and hypotension: One of the causes and one of the consequences of poor tolerance of intermittent hemodialysis in the ICU?

**DOI:** 10.1186/s13054-016-1331-4

**Published:** 2016-06-10

**Authors:** Jérôme Allyn, Laure Corradi, Nicolas Allou, Bernard Alex Gaüzère

**Affiliations:** Intensive Care Unit, Centre Hospitalier Universitaire de La Réunion, Hôpital Félix Guyon, 97405 Saint-Denis, France; Centre René Labusquière, Université de Bordeaux, 33076 Bordeaux cedex, France

In the context of a better management of a negative fluid balance in critically ill patients, Bitker et al. recently published a prospective, observational study in *Critical Care* [1]. The occurrence of hypotension in 47 patients during 107 sessions of intermittent hemodialysis (IHD) was analyzed. The authors demonstrated that hypotension occurs frequently (57 %) but is seldom linked with a preload dependence defined by a passive leg raising test (cardiac index monitoring by a PiCCO® device; Pulsion Medical Systems, Feldkirchen, Germany). Although this study clarifies part of the genesis of hypotension during IHD, some comments can be made.

First, the authors focused on the occurrence of hypotensive episodes (an episode of systolic arterial pressure below 65 mm Hg, without duration criterion, and only the first episode of hypotension) during the IHD session. It is indeed a frequent complication that compromises the IHD session and the volume of fluid removal [2]. We question the clinical relevance of this criterion; it would have been useful to record other complications of IHD in hypovolemic patients, like supraventricular arrhythmias, elevation of the lactate level, or increase of vasopressor doses.

Second, the authors focused on preload dependence as the cause of hypotension during the IHD session, whereas many other factors such as vasoplegia or induced cardiac depression may be involved [2]. Therefore, an echocardiographic assessment before and during the episode of hypotension could have been very helpful to explore the different causes of hypotension. The value of the mitral inflow E wave to early diastolic mitral annulus velocities ratio (E/Ea ratio) is a marker of the left ventricular filling pressure which was well validated by Vignon et al. in a study of hemodialysis-induced preload reduction [3] and which has already been linked to poor tolerance of fluid removal led by furosemide or hemofiltration (area under receiver operating characteristic curves of 0.74) [4]. One study failed to identify echocardiographic parameters linked with a poor tolerance of an IHD session, but its statistical power was quite low [5].

Last, the authors propose PiCCO® parameters as predictors for hypotension associated with preload dependence during IHD in critically ill patients. Maybe a strictly non-invasive monitoring, like echocardiography, should be preferred to the PiCCO® system at this late stage of treatment (median of 18 days). Nevertheless, Bitker et al*.* demonstrated that the first hypotensive episode during IHD sessions is rarely associated with a preload dependence.

## Abbreviations

E/Ea: mitral inflow E wave to early diastolic mitral annulus velocities ratio
ICU: intensive care unit
IHD: intermittent hemodialysis
