# Supplementary material for: Preload dependence and hypotension: One of the causes and one of the consequences of poor tolerance of intermittent hemodialysis in the ICU?
Source: Crit Care. 2016 Jun 10;20:155. doi: 10.1186/s13054-016-1331-4 (PMC4901395; doi:10.1186/s13054-016-1331-4)
Supplement: Related texts

# Reply: Authors’ response

**Authors:** Laurent Bitker, Jean-Christophe Richard

In their letter, Allyn et al. raised interesting issues regarding our study assessing the prevalence of preload-dependent hypotension during IHD in the intensive care unit (ICU) [1]. We acknowledged the absence of a universally accepted definition of hemodynamic tolerance and the variability of criteria found in the literature. However, we decided to identify hemodynamic intolerance by a mean arterial pressure lower than 65 mm Hg, as it is a widespread criterion used in everyday practice to adjust catecholamine dose. Nevertheless, our risk factors for hemodynamic tolerance during IHD were similar to those reported in a recent study [6], suggesting that slight differences in definition of IHD hemodynamic intolerance have little impact on study results.

Allyn et al. state their preference for echocardiography over PiCCO® to assess hemodynamic tolerance during IHD. Although we acknowledge that the two techniques may be interchangeable or complementary or both, the PiCCO® device allows continuous hemodynamic monitoring during IHD and may be used more easily.

Vignon et al. showed that the mitral inflow wave to early diastolic septal mitral annulus velocities ratio (E/E′) was related to fluid removal and cardiac preload during IHD [3]. In a selected population of 40 hemodynamically stable, ventilated acute respiratory distress syndrome patients in whom a negative fluid balance strategy could be applied, E/E′ was indeed a satisfactory predictor of poor hemodynamic tolerance to fluid depletion [4], although its relatively low specificity (0.46) suggests that factors unrelated to cardiac preload were involved. Still, another study of 54 unselected ICU patients failed to identify any echocardiographic parameter related to cardiac preload as predictors of hemodynamic tolerance of IHD [5]. In our study [1], preload dependence at IHD onset was not predictive of hypotension during IHD, and poor hemodynamic tolerance of IHD was rarely related to preload dependence, essentially in mechanically ventilated patients.

Altogether, these findings might suggest that assessment of cardiac preload before IHD (assessed by any monitoring device) predicts hemodynamic intolerance of IHD in specific ICU populations (mechanically ventilated and hemodynamically stable patients), although the specific mechanism of hypotension should prompt an evaluation of hemodynamic status (by echocardiography, PiCCO®) to re-evaluate fluid removal rate by IHD.
